# Supplementary material for: Running wheel exercise induces therapeutic and preventive effects on inflammatory stimulus-induced persistent hyperalgesia in mice
Source: PLoS One. 2020 Oct 13;15(10):e0240115. doi: 10.1371/journal.pone.0240115 (PMC7553300; doi:10.1371/journal.pone.0240115)
Supplement: Attachment — Submitted filename: PLOS ONE REVIEW 2020.docx [file pone.0240115.s001.docx]

ID: PONE-D-20-03419

These investigators injected the inflammatory mediator PGE2 or vehicle intraplantarly into the hindpaw of mice on a daily basis for 14 days and then at day 14 the injections were stopped and running wheels (or no wheel controls) were introduced to the mouse cages for 28 days. Mice were tested weekly for hindpaw von Frey hyperalgesia and at 7 and 14 days of PGE2 injection the mice had hyperalgesia. Having free access to the exercise wheel partially reduce hyperalgesia after 7, 14, 21, and 28 days of exercise. There were no differences in daily running distance between the vehicle and PGE2 injected mice. Capsaicin intraplantar injection caused increased flinching in no wheel/no PGE treatment group compared to vehicle treated or PGE2/wheel treated mice after 28 days of wheel access. These results indicate an analgesic effect of wheel exercise on PGE2 induced chronic von Frey hyperalgesia and capsaicin induced flinching, but not on daily distance running.

Another experiment started with a 14 day course of daily PGE2 or vehicle intraplantar injections, then 14 days of running wheel access or no wheel access. There was no difference in the distance run/day between PGE2 and vehicle. Von Frey hindpaw threshold testing was performed weekly during the entire time period and for 14 days after the wheel access ended. The PGE2 mice developed von Frey hyperalgesia by day 7 of injections and wheel exercise starting at the end of the PGE2 treatment partially reversed this hyperalgesia and this effect persisted for 14 days after stopping wheel exercise. Capsaicin intraplantar injection caused no increased flinching in PGE2/wheel treated mice 14 days after wheel access.

Another experiment started with a 14 day course of daily PGE2 or vehicle intraplantar injections, then 14 days of no treatment, then 14 days of running wheel access or no wheel access. There was no difference in the distance run/day between PGE2 and vehicle. The PGE2 mice developed von Frey hyperalgesia by day 7 of injections and wheel exercise starting at 14 days after the end of the PGE2 treatment partially reversed this hyperalgesia. Capsaicin intraplantar injection caused no increased flinching PGE2/wheel treated mice after 14 days of wheel access.

Another experiment provided the mice with 28 days wheel access or no wheel access, starting at day 1, then after 8 days of wheel or no wheel access PGE2 or vehicle daily intraplantar injections were started for 14 days. The PGE2 mice did display a reduction in daily wheel running distance relative to the vehicle group. The PGE2/no wheel mice developed von Frey hyperalgesia by day 7 of PGE2 injections, but the PGE2/ wheel exercise group did not develop hyperalgesia. Capsaicin intraplantar injection caused no increased flinching PGE2/wheel treated mice after 14 days of wheel access.

Theses results are novel and the experiments seem to be well performed but there are several issues.

Major Concerns:

1. Repeated testing over time within the same groups of mice need to be analyzed	with repeated measures 2 way ANOVA with Sidak post hoc testing.

2 The introduction and discussion need extensive revision with an English	speaking editor.

3. There are many paragraphs in the introduction and discussion that are	tangential or do not add to hypothesis or data, would recommend deleting lines	45-59, 76-78, 384-393, 418-422, 459-470.

4. There is no discussion regarding the mechanisms by which repeated PGE2	injections cause long lasting hyperalgesia. Dr Parada coauthored many papers on	this topic with Dr Ferreira and it is surprising there is no discussion on how PGE2	injections cause long acting hyperalgesia by up-regulating the Nav1.8 sodium	channels in the DRG, that PGE2 hyperalgesia is mediated by nonpeptidergic	small sensory neurons that normally do not contribute to von Frey thresholds, and	most importantly, that PGE2 induced mechanical hyperalgesia is mediated by	DRG glutamate release acting on the NMDA receptor on DRG satellite cells.	DRG satellite cells are immune cells capable of releasing proinflammatory	cytokines and growth factors that may mediate chronic PGE2 sensitization.

5. The discussion of the prior literature on exercise analgesic mechanisms in	rodent pain models is superficial and incomplete. More discussion of exercise	suppression of pronociceptive immune responses is required and this should be	tied into the crucial role of DRG satellite immune cells in PGE2 chronic	hyperalgesia. The following papers should be included and reviewed; Lopez-	Alvarez et al, Pain 2015: 156: 1812-25; Almeida et al, Pain 2015:156:504-	513;	Cobianchi et al, Neuroscience 2010: 168: 273-287; Shi et al, Anesthesiology	2018: 129: 557-75.

6. This is a descriptive paper with no mechanism or hypothesis of a mechanism	for the analgesic effects of exercise.

Minor concerns:

1. Need to make clear in methods if mice were housed in individual cages and if they had the wheel in their cage 24 hours/day.
2. There is no information on the running wheels, vendor, or computerized data collection methods for measuring distance run/day?
3. Why did the authors inject the same amount and volume of PGE2 in mice as they previously used in rats, shouldn’t smaller doses and volumes be used in much smaller animals, please add to the discussion in a section on the limitations of the study.
4. All figure legends need to be on one page.
