# Supplementary material for: Running wheel exercise induces therapeutic and preventive effects on inflammatory stimulus-induced persistent hyperalgesia in mice
Source: PLoS One. 2020 Oct 13;15(10):e0240115. doi: 10.1371/journal.pone.0240115 (PMC7553300; doi:10.1371/journal.pone.0240115)
Supplement: Attachment — Submitted filename: Response to Reviewers.docx [file pone.0240115.s002.docx]

Authors: First, we would like to thank the contributions made by the editor and reviewer #1. Below are our comments, point-by-point, to the comments and questions raised. We are at your disposal for any additional clarification that may be required.

**#Editor:** *“Dear Dr. Sartori,

Thank you for submitting your manuscript to PLOS ONE. After careful consideration, we feel that it has merit but does not fully meet PLOS ONE’s publication criteria as it currently stands. Therefore, we invite you to submit a revised version of the manuscript that addresses the points raised during the review process.*

*Only one of the original reviewers were available to review your revision (see below for their comments). Both the reviewer and I agree that the manuscript reports a series of interesting studies. However, the Discussion lacks some critical view and insights into possible mechanisms. Currently the Discussion is mostly repetition of the results and how the results confirm some of the pastliterature. This does not meet the scientific standards required in PLOS ONE. Therefore, I would like to encourage authors to discuss possible mechanisms underlying their findings.”*

Authors: We appreciate the comment and, in the revised manuscript, we added some critical view and insights into possible mechanism behind our findings. We better discussed the anti-inflammatory effect of physical exercise and its effects on endogenous PGE_2_. Also, we discussed the possible involvement of the serotonergic and opioidergic system in the exercise-induced analgesia. We also improved the discussion regarding the effects of exercise on DRG. Finally, we highlighted the effect of exercise on oxidative stress and the endocannabinoid system as possible mechanisms.

*“Additionally, as the authors acknowledge that the molecular mechanisms is important, please include this in your discussion.”*

Authors: We included this statement in the revised manuscript.

*“Please submit your revised manuscript by Sep 26 2020 11:59PM. If you will need more time than this to complete your revisions, please reply to this message or contact the journal office at plosone@plos.org. When you're ready to submit your revision, log on to https://www.editorialmanager.com/pone/ and select the 'Submissions Needing Revision' folder to locate your manuscript file.

Please include the following items when submitting your revised manuscript:*

- *A rebuttal letter that responds to each point raised by the academic editor and reviewer(s). You should upload this letter as a separate file labeled 'Response toReviewers'.*
- *A marked-up copy of your manuscript that highlights changes made to the original version. You should upload this as a separate file labeled 'Revised Manuscript with Track Changes'.*
- *An unmarked version of your revised paper without tracked changes. You should upload this as a separate file labeled 'Manuscript'.*

*If you would like to make changes to your financial disclosure, please include your updated statement in your cover letter. Guidelines for resubmitting your figure files are available below the reviewer comments at the end of this letter.

If applicable, we recommend that you deposit your laboratory protocols in protocols.io to enhance the reproducibility of your results. Protocols.io assigns your protocol its own identifier (DOI) so that it can be cited independently in the future. For instructions see:* [*http://journals.plos.org/plosone/s/submission-guidelines#loc-laboratory-protocols*](http://journals.plos.org/plosone/s/submission-guidelines#loc-laboratory-protocols) *We look forward to receiving your revised manuscript.

Kind regards,

Amir-Homayoun Javadi, PhD
Academic Editor
PLOS ONE”*

Authors: Appreciated.

**--------------------------------------------------------------------------------------------------------------------------------------------**

*“Reviewers' comments:

Reviewer's Responses to Questions*

***Comments to the Author*** *1. If the authors have adequately addressed your comments raised in a previous round of review and you feel that this manuscript is now acceptable for publication, you may indicate that here to bypass the “Comments to the Author” section, enter your conflict of interest statement in the “Confidential to Editor” section, and submit your "Accept" recommendation.*

*Reviewer#1: All comments have been addressed”*

Authors: Appreciated

*“2. Is the manuscript technically sound, and do the data support the conclusions?

The manuscript must describe a technically sound piece of scientific research with data that supports the conclusions. Experiments must have been conducted rigorously, with appropriate controls, replication, and sample sizes. The conclusions must be drawn appropriately based on the data presented.*

*Reviewer #1: No”*

Authors: In the revised manuscript we made few changes in our conclusion to directly refer to our findings.

*“3. Has the statistical analysis been performed appropriately and rigorously?*

*Reviewer #1: Yes”*

Authors: Appreciated

*“4. Have the authors made all data underlying the findings in their manuscript fully available?

The* [*PLOS Data policy*](http://www.plosone.org/static/policies.action#sharing) *requires authors to make all data underlying the findings described in their manuscript fully available withoutrestriction, with rare exception (please refer to the Data Availability Statement in the manuscript PDF file). The data should be provided as part of the manuscript or its supporting information, or deposited to a public repository. For example, in addition to summary statistics, the data points behind means, medians and variance measures should be available. If there are restrictions on publicly sharing data—e.g. participant privacy or use of data from a third party—those must be specified.*

*Reviewer #1: No”*

Authors: There is a statement declaring that “All data presented in the current manuscript can be obtained from the corresponding author”.

*“5. Is the manuscript presented in an intelligible fashion and written in standard English?

PLOS ONE does not copyedit accepted manuscripts, so the language in submitted articles must be clear, correct, and unambiguous. Any typographical or grammatical errors should be corrected at revision, so please note any specific errors here.*

*Reviewer #1: Yes”*

Authors: Appreciated

*“6.Review Comments to the Author

Please use the space provided to explain your answers to the questions above. You may also include additional comments for the author, including concerns about dual publication, research ethics, or publication ethics. (Please upload your review as an attachment if it exceeds 20,000 characters)*

*Reviewer #1: (No Response)”*

Authors: Appreciated

*“7. PLOS authors have the option to publish the peer review history of their article (*[*what does this mean?*](https://journals.plos.org/plosone/s/editorial-and-peer-review-process#loc-peer-review-history)*). If published, this will include your full peer review and any attached files.


If you choose “no”, your identity will remain anonymous but your review may still be made public.*


***Do you want your identity to be public for this peer review?*** *For information about this choice, including consentwithdrawal, please see our* [*Privacy Policy*](https://www.plos.org/privacy-policy)*.*

*Reviewer #1: No”*

Authors: Appreciated
